# Gut Microbiota Alterations in Hypothyroidism: A Pilot Study Revealing Increased Abundance of Specific Bacterial Genera

**DOI:** 10.1155/jnme/9988966

**Published:** 2026-02-27

**Authors:** Zahra Hoseini Tavassol, Farima Farsi, Fateme Ettehad-Marvasti, Hanieh-Sadat Ejtahed, Shirin Hasani-Ranjbar

**Affiliations:** ^1^ Obesity and Eating Habits Research Center, Endocrinology and Metabolism Clinical Sciences Institute, Tehran University of Medical Sciences, Tehran, Iran, tums.ac.ir; ^2^ Endocrinology and Metabolism Research Center, Endocrinology and Metabolism Clinical Sciences Institute, Tehran University of Medical Sciences, Tehran, Iran, tums.ac.ir

**Keywords:** gut microbiota, hypothyroidism, polymerase chain reaction

## Abstract

**Background:**

Hypothyroidism (HT) is a prevalent thyroid disorder characterized by insufficient thyroid hormone production, leading to metabolic complications. Emerging research suggests a link between gut microbiota and thyroid regulation, positing that alterations in gut bacterial populations may contribute to HT’s development and progression. This study aimed to investigate these associations by comparing gut microbiota compositions between individuals with HT and healthy adults, potentially refining diagnostic tools and therapeutic strategies.

**Methods:**

In this pilot study conducted between 2019 and 2023, 15 hypothyroid patients and 15 age‐ and gender‐matched healthy controls participated in the study. Exclusion criteria were applied to eliminate confounding factors. Anthropometric data were collected, and stool samples underwent microbial analysis. Total bacterial DNA was extracted, and quantitative real‐time PCR targeting 16S rRNA genes across eight bacterial genera was performed. The Mann–Whitney *U* test was used for statistical analyses.

**Results:**

No significant differences were observed in baseline demographic and anthropometric characteristics between groups. However, hypothyroid patients exhibited significantly elevated levels of *Bacteroides*, *Bifidobacterium*, *Escherichia*, *Fecalibacterium*, and *Prevotella* (*p* values < 0.001–0.030). No significant differences were found in levels of *Akkermansia, Lactobacillus*, or in the *Bacteroides*/*Prevotella* ratio.

**Conclusion:**

This pilot study provides preliminary indications of a possible role of gut microbiota in the pathophysiology of HT. Variations in bacterial composition suggest a significant influence of gut health on thyroid regulation. Future studies with larger cohorts are needed to explore the biological pathways linking the gut microbiome to thyroid function, which may lead to novel microbiota‐targeted therapeutic approaches.

## 1. Introduction

Thyroid disorders are widely recognized as significant risks to metabolic health [1], necessitating accurate diagnosis and effective treatment strategies. Achieving these objectives requires a comprehensive understanding of the various factors contributing to these conditions.

The relationship between gut microbiota and thyroid homeostasis has gained substantial recognition in recent scientific literature, with disruptions in microbial balance—commonly termed dysbiosis—being implicated in a variety of thyroid‐related disorders. Dysbiosis can compromise the intestinal barrier, a fundamental component of immune regulation. When this barrier is impaired, there is an elevated risk of immune and metabolic disturbances that may affect the body systematically. These gut‐related disruptions are now associated with the pathogenesis of multiple thyroid conditions, including autoimmune thyroiditis, hypothyroidism (HT), and hyperthyroidism. Current research underscores the critical need for a balanced gut microbiome to maintain both digestive health and thyroid function, positioning the microbiome as an essential player in immune system modulation and endocrine stability [2–6].

Recent research suggests that gut microbiota profoundly impact health by modifying hormone structures and transport pathways, influencing the bioavailability of key micronutrients needed for hormone synthesis, and adjusting immune responses to self‐antigens. These interactions underscore the microbiome’s crucial role in maintaining hormonal balance and supporting immune stability, highlighting its importance in broader physiological health [7–9].

HT, characterized by insufficient thyroid hormone production, is commonly associated with various metabolic disruptions [10]. Research increasingly suggests that bacteria capable of producing short‐chain fatty acids (SCFAs)—including acetate, propionate, and butyrate—may aid in hormonal regulation. These SCFAs may enhance the activity of triiodothyronine (T3) through mechanisms affecting the anterior pituitary [11, 12]. Hashimoto’s thyroiditis, an autoimmune disorder and a primary cause of HT, triggers the immune system to produce specific antithyroid antibodies, such as anti‐thyroid peroxidase (anti‐TPO), which ultimately interfere with thyroid function [10–13]. While the precise origins of HT remain incompletely understood, disruptions in gut microbiota, alongside genetic and environmental influences, are increasingly recognized as contributing factors. Studies have observed that individuals with HT exhibit shifts in gut microbiota composition that correspond with clinical markers of the disease [14–16]. Moreover, HT frequently coincides with small intestinal bacterial overgrowth, potentially related to the reduced intestinal motility characteristic of the condition [2]. Further research has shown that certain strains of *Bifidobacterium* and *Lactobacillus* contain unique amino acid sequences capable of selectively binding to human autoantibodies directed at thyroid peroxidase and thyroglobulin [17]. This finding suggests that gut microbiota composition may play a role in the clinical manifestations of HT, could potentially inform future adjunctive therapeutic strategies, and may even hold prognostic value in identifying patients at risk of disease progression or treatment response variability. The present study aims to expand on these findings by comparing primary gut bacterial profiles in hypothyroid patients and healthy controls, providing further insight into these potential microbiota‐related associations. As existing findings remain inconsistent and exploratory, this study was designed as a pilot investigation to generate preliminary data and guide future adequately powered research.

## 2. Methods

### 2.1. Study Design

This pilot study recruited 15 patients diagnosed with HT and 15 control participants between 2019 and 2023 at Shariati Teaching Hospital in Tehran, Iran. Overt primary HT was confirmed based on elevated serum thyroid‐stimulating hormone (TSH) levels together with reduced free thyroxine (FT4) levels, in accordance with standard clinical recommendations for diagnosing HT [18]. Diagnosis was verified using medical records. None of the hypothyroid patients had a history of thyroid surgery, central (secondary) HT, or medication‐induced HT. The case group included both anti‐TPO–positive and anti‐TPO–negative individuals. Detailed etiological classification and precise disease duration were not consistently available for all participants and were therefore not analyzed. Healthy controls were recruited from the same center and had no known history of thyroid disease or any chronic systemic illness based on clinical interview and review of medical records.

The study excluded participants who were pregnant or lactating, as well as individuals who were smokers or had used corticosteroids, antibiotics, prebiotics, or probiotics within 3 months prior to the study. Additional exclusion criteria encompassed comorbid conditions such as cardiovascular disease, renal or liver disorders, gastrointestinal diseases, inflammatory bowel disease, cancer, or any history of acute or chronic diarrhea within the past month. All procedures were conducted in compliance with ethical standards and were approved by the Ethics Committee of the National Institute for Medical Research Development (NIMAD) (ID number: IR.NIMAD.REC.1398.152). Written informed consent was obtained from all participants.

### 2.2. Measurements

Baseline characteristics of the participants, including age and gender, were recorded alongside key anthropometric measurements. These measurements—weight (taken with a digital scale by Seca, Germany), height (measured using a stadiometer), body mass index (BMI), waist circumference (WC), and hip circumference (HC)—were systematically documented. Thyroid blood test results were obtained directly from patient medical records. Thyroid function tests (TSH and FT4) were used to confirm the diagnosis of HT and to verify normal thyroid status in healthy controls. Basic information on levothyroxine treatment status was available; however, detailed data on dosage and disease duration were not consistently documented for all hypothyroid patients and were therefore not included as baseline characteristics. For microbial analysis, fresh stool samples were collected in sterile containers, promptly placed on ice packs, and transported to the lab within 2 hours. Upon arrival, the samples were frozen at −80°C to ensure preservation for later microbial testing. Each 200 mg sample underwent total bacterial DNA extraction using the QIAamp DNA Stool Mini Kit (QIAGEN, Germany) according to manufacturer instructions. To assess DNA purity and concentration, a NanoDrop spectrophotometer (Thermo Scientific NanoDrop, USA) was used. All extracted DNA samples were then stored at −20°C until further analysis. Eight genera were selected a priori based on previous evidence implicating them in thyroid‐related immune or metabolic pathways, alterations in thyroid disease or gut‐thyroid axis dysfunction [16, 19, 20].

Quantitative real‐time PCR was conducted in triplicate for hypothyroid samples on the Applied Biosystems platform (Foster City, CA) and for control samples using the Roche LightCycler 96 System (Switzerland). For amplification, genus‐specific primers were employed to target bacterial 16S rRNA genes of *Akkermansia*, *Bacteroides*, *Bifidobacterium*, *Escherichia*, *Fecalibacterium*, *Lactobacillus*, and *Prevotella* [21–27]. Table 1 presents a summary of selected taxa characterized in the gut microbiota by real‐time PCR detection and quantification with 16S rRNA gene‐targeted primers. The primers with their respective amplicon sizes used in this study were selected based on previously published and validated assays. In our laboratory, primer specificity was confirmed by melt‐curve analysis and the absence of nonspecific amplification, while amplification efficiencies followed the patterns reported in the original validation studies. Schneeberger et al. (2015) described the *Akkermansia muciniphila* primers. Their development specifically aimed to target this bacterium, driven by its ubiquitous role in mediating inflammation and regulating adipose tissue homeostasis, as the *A. muciniphila* levels were decreased in metabolic dysregulation features. The Bacteroides primers were also modified from Gregory et al. The study was key research in this field, highlighting the significance of colonization of the infant gut in the early period, which serves as a crucial stage for immune system development and pathogen defense, making it a key model for microbiome studies. For *Bifidobacterium*, the primer set reported by Rinttilä et al. remains the gold standard in microbiome analysis due to its proven high specificity and sensitivity across multiple population contexts. *Escherichia* spp*.* Primers were utilized from Bartosch et al. These primers are especially useful in examining cognitive disturbances associated with changes in gut microbiota during aging or antibiotic treatment. The *Fecalibacterium prausnitzii* primers described by Fitzgerald et al. were used thanks to their role in identifying the anti‐inflammatory bacterium. This study further highlights the genomic plasticity of *F. prausnitzii* and its relevance in vitro. In a similar manner, the primers reported by Kanno et al. were chosen because they have been shown to be able to detect changes in intestinal flora caused by decreased gastric acid. Lastly, *Prevotella* primers, from Larsen et al., were selected because this bacterium may contribute to the microbiota changes that could lead to metabolic diseases such as type 2 diabetes. Together, this primer suite enables high‐throughput, reproducible, and informative profiling of gut microbiota. The PCR thermal cycling process began with an initial DNA denaturation at 95°C for 3 min, followed by 40 cycles: 5 s of denaturation at 95°C, 30 s of annealing at 55°C, and 30 s of extension at 72°C. To confirm amplification specificity, a melting curve analysis was performed by gradually cooling the PCR from 95°C to 60°C. Bacterial concentrations in each sample were quantified by comparing threshold cycle (Ct) values to standard curves, which were created for each experiment using serial 10‐fold dilutions of *Escherichia coli* genomic DNA (ATCC 25922) with known concentrations. We acknowledge that using *E. coli* genomic DNA as a universal standard does not account for genus‐specific differences in 16S rRNA gene copy number or genome size; however, genus‐specific DNA standards were not available. Therefore, the obtained bacterial quantities represent relative estimations rather than absolute copy number equivalents for each genus. Finally, bacterial copy numbers for each of the eight bacterial genera were calculated on a per gram basis of feces. Because qPCR quantifies 16S rRNA gene copies rather than viable colony‐forming units, the resulting values represent gene copy numbers per gram of stool.

**TABLE 1:** 16S rRNA gene‐targeted specific primers used for real‐time PCR.

Target bacteria	Oligonucleotide sequence	Amplicon size (bp)	Reference
*Akkermansia*	F: CAG​CAC​GTG​AAG​GTG​GGG​AC R: CCT​TGC​GGT​TGG​CTT​CAG​AT	329	Schneeberger (2015) [21]
*Bacteroides*	F: GGT​GTC​GGC​TTA​AGT​GCC​AT R: CGGAYGTAAGGGCCGTGC	140	Gregory (2015) [22]
*Bifidobacterium*	F: TCGCGTCYGGTGTGAAAG R: CCACATCCAGCRTCCAC	243	Rinttila (2004) [23]
*Escherichia*	F: CAT​TGA​CGT​TAC​CCG​CAG​AAG​AAG​C R: CTC​TAC​GAG​ACT​CAA​GCT​TGC	190	Bartosch (2004) [24]
*Fecalibacterium*	F: GGA​GGA​AGA​AGG​TCT​TCG​G R: AAT​TCC​GCC​TAC​CTC​TGC​ACT	248	Fitzgerald (2018) [25]
*Lactobacillus*	F: AGC​AGT​AGG​GAA​TCT​TCC​A R: CACCGCTACACATGGAG	341	Kanno (2009) [26]
*Prevotella*	F: CACCAAGGCGACGATCA R: GGATAACGCCYGGACCT	283	Larsen (2010) [27]

### 2.3. Use of Artificial Intelligence Tools

The scientific writing, data analysis, and interpretation in this publication did not use any content created by artificial intelligence. Large language model tools were solely used for language editing during the revision process. The authors examined and approved all the content.

### 2.4. Statistical Analysis

Statistical analyses were conducted using SPSS software, Version 22.0 (SPSS Inc., Chicago, IL, USA). The normality of variable distributions was assessed using the Kolmogorov–Smirnov test. Data are presented as the median ± interquartile range. To compare data between the hypothyroid and control groups, the Mann–Whitney *U* test was applied, with a significance threshold set at a *p*‐value of < 0.05. Given the exploratory pilot design and limited sample size, statistical findings should be interpreted as preliminary.

## 3. Result

This pilot analysis provides a comparison of baseline characteristics and anthropometric measurements between participants diagnosed with HT (*n* = 15) and a matched control group of healthy individuals (*n* = 15), presented in Table 2. The hypothyroid group consisted primarily of female participants (*n* = 13), with only two males, while the control group was entirely female (*n* = 15). The median age showed a slight difference, with the hypothyroid group having a median age of 29 years (interquartile range: 24.5–48.5) compared to 38 years (interquartile range: 33.5–42.5) in the control group; however, this difference was not statistically significant (*p* = 0.555). Anthropometric measurements were also comparable between the groups. The median weight for the hypothyroid group was 74.0 kg (interquartile range: 52.5–90.0), versus 82.5 kg (interquartile range: 77.5–84.8) for controls, with no statistically significant difference (*p* = 0.317). Heights were nearly the same, with a median of 164.0 cm (interquartile range: 163.0–167.8) in the hypothyroid group and 163.0 cm (interquartile range: 156.8–165.3) among controls (*p* = 0.459). BMI values followed a similar trend, with the hypothyroid group having a median BMI of 25.6 kg/m^2^ (interquartile range: 21.4–33.8) compared to 31.6 kg/m^2^ (interquartile range: 30.8–32.2) in the control group, which was also not statistically significant (*p* = 0.446). WC and HC measurements showed no notable differences between groups. The hypothyroid group had a median WC of 94.0 cm (interquartile range: 77.0–100.5), while the control group had a median of 100.0 cm (interquartile range: 94.0–104.0), with a *p*‐value of 0.123. HC medians were 100.0 cm (interquartile range: 92.5–118.0) for the hypothyroid group and 114.5 cm (interquartile range: 113.0–116.0) for the control group, yielding a *p*‐value of 0.298. Collectively, these results indicate that there were no statistically significant differences in anthropometric measurements between the hypothyroid and control groups.

**TABLE 2:** Participants’ characteristics and baseline measurements.

	**Hypothyroidism, N = 15 (13 females)**	**Healthy control, N = 15 (15 females)**	**p** **value**

Age (years)	29.00 (24.50–48.50)	38.00 (33.50–42.50)	0.555
Weight (kg)	74.00 (52.50–90.00)	82.50 (77.50–84.75)	0.317
Height (cm)	164.00 (163.00–167.75)	163.00 (156.75–165.25)	0.459
BMI (kg/m^2^)	25.60 (21.40–33.77)	31.61 (30.75–32.17)	0.446
WC (cm)	94.00 (77.00–100.50)	100.00 (94.00–104.00)	0.123
HC (cm)	100.00 (92.50–118.00)	114.53 (113.00–116.00)	0.298

*Note:* Comparisons were made with Mann–Whitney. Values are stated as median (interquartile range). *p* value < 0.05 was considered statistically significant.

Abbreviations: BMI, body mass index; HC, hip circumference; WC, waist circumference.

Table 1 summarizes the 16S rRNA gene‐targeted primers used in real‐time PCR to detect and quantify specific bacterial taxa within the gut microbiota. Each primer set is uniquely tailored to amplify a particular bacterial group, with precise sequences specified for both the forward (F) and reverse (*R*) primers and corresponding amplicon sizes. For instance, primers designed for *Akkermansia* produce a 329 bp amplicon, as detailed by Schneeberger et al. In contrast, primers for *Bacteroides* and *Bifidobacterium* yield amplicons of 140 bp and 243 bp, respectively, based on sequences reported by Gregory and Rinttila. *Escherichia* primers result in a 190 bp amplicon, following Bartosch’s published sequences. Primers for *Fecalibacterium* and *Lactobacillus* produce amplicons of 248 bp and 341 bp, as described by Fitzgerald and Kanno, respectively. Lastly, primers for *Prevotella* generate a 283 bp amplicon, according to Larsen. This carefully curated set of primers allows for precise identification and quantification of different bacterial groups within the gut microbiota, thereby enhancing the reliability and accuracy of microbiome analysis.

A comparative analysis of fecal microbiota composition between hypothyroid patients and healthy controls, quantified in log10 gene copies per gram of stool, is shown in Table 3. The analysis reveals significant differences in bacterial taxa, indicating distinct shifts in gut microbiota linked to HT. *Akkermansia* levels were notably higher in the control group, with a median of 4.2300 (interquartile range: 2.6051–4.7237), compared to a median of −0.2301 (interquartile range: −1.4219–4.9253) in the hypothyroid group; however, this difference was not statistically significant (*p* = 0.061). The negative log10 values observed for *Akkermansia* reflect extremely low but detectable gene copy numbers that were below 1 copy per gram of stool, which yield negative values when log10‐transformed. Conversely, *Bacteroides* showed a significantly higher abundance in hypothyroid patients, with a median of 17.9682 (interquartile range 15.9909–20.2539) compared to 7.4778 (interquartile range: 6.6639–7.7380) in the control group (*p* < 0.001). Similar trends were observed for *Bifidobacterium* and *Escherichia*, both of which were significantly elevated in hypothyroid individuals, with *p*‐values of 0.030 and 0.007, respectively. *Fecalibacterium* levels also differed markedly, with hypothyroid patients showing a median of 12.1218 (interquartile range: 11.3637–13.6283) compared to 6.7132 (interquartile range: 5.4876–7.3813) in the controls (*p* < 0.001). No significant difference was observed in *Lactobacillus* levels between the two groups (*p* = 0.343). However, *Prevotella* was considerably more abundant in the hypothyroid group (*p* = 0.002). Interestingly, despite these shifts, the *Bacteroides*/*Prevotella* ratio remained comparable across groups, showing no statistically significant difference (*p* = 0.739). Overall, these findings underscore distinctive microbiota alterations in hypothyroid patients, particularly with elevated levels of *Bacteroides*, *Bifidobacterium*, *Escherichia*, *Fecalibacterium*, and *Prevotella*.

**TABLE 3:** Comparison of the fecal microbiotas of hypothyroidism patients and healthy controls.

	Hypothyroidism	Healthy control	*p* value
*Akkermansia*	−0.2301 (−1.4219–4.9253)	4.2300 (2.6051–4.7237)	0.061
*Bacteroides*	17.9682 (15.9909–20.2539)	7.4778 (6.6639–7.7380)	< 0.001
*Bifidobacterium*	7.2776 (4.3960–8.1852)	4.7184 (3.6187–5.1010)	0.030
*Escherichia*	6.1782 (4.7415–8.2516)	4.1382 (3.2433–5.0611)	0.007
*Fecalibacterium*	12.1218 (11.3637–13.6283)	6.7132 (5.4876–7.3813)	< 0.001
*Lactobacillus*	6.4391 (0.3066–8.5404)	4.0379 (3.7472–4.7596)	0.343
*Prevotella*	15.2891 (10.5196–17.0256)	5.0505 (2.9174–8.3582)	0.002
*Bacteroides*/*Prevotella* ratio	1.1874 (1.1360–1.7823)	1.2305 (0.9345–2.3230)	0.739

*Note:* All values were calculated based on the log10 gene copies/gram stool unit. Values are stated as median (interquartile range). Comparisons were made with Mann–Whitney. *p* value < 0.05 was considered statistically significant.

## 4. Discussion

HT is among the most prevalent thyroid disorders, often accompanied by various complications, with autoimmune HT recognized as the most common autoimmune disorder globally [28]. Understanding the etiology and identifying related factors are essential for improving treatment strategies for this condition.

This study is the first in Iran to examine the association between HT and changes in gut microbiota. This pilot study suggests higher levels of *Bacteroides*, *Bifidobacterium*, *Escherichia*, *Fecalibacterium*, and *Prevotella* in hypothyroid patients compared to healthy controls, while *Akkermansia* and *Lactobacillus* levels showed no significant differences. The *Bacteroides*/*Prevotella* ratio was also similar between the groups. While several taxa showed visually striking numerical differences between groups, the magnitude of these differences should not be overinterpreted. In this pilot study, qPCR‐derived gene copy numbers represent relative estimates influenced by technical and biological variability and do not directly translate into effect size or functional impact. Therefore, the results are best interpreted as preliminary signals warranting confirmation in larger, well‐powered studies. It is also important to emphasize that our analysis was limited to eight preselected bacterial genera, and therefore the findings should not be interpreted as representing global gut microbiota alterations.

Fuya Zhao et al. observed notable shifts in gut microbiota among hypothyroid individuals. Their study found decreased levels of specific genera, including *Bacteroides*, *Fecalibacterium*, *Prevotella*_9, and *Lachnoclostridium*, in hypothyroid patients, while other genera—such as *Blautia*, *Ruminococcus torques* group, *Roseburia*, *Fusicatenibacter*, *Romboutsia*, *Dorea*, and *Eubacterium hallii* group—were increased. These findings suggest a complex interplay between HT and gut microbiota, potentially affecting the condition’s progression and management [15]. In contrast, our study observed elevated levels of *Fecalibacterium* and *Prevotella* among hypothyroid patients. Such discrepancies may reflect natural variations in microbiota profiles due to geographical, dietary, or genetic factors, underscoring the complexity of the HT‐microbiota relationship and suggesting it may not be consistent across different populations.

According to the study conducted by Simo Liu et al., hypothyroid patients exhibited reduced bacterial diversity, with a noted increase in *Phascolarctobacterium*. The authors proposed that *Phascolarctobacterium* might be linked to disruptions in metabolic pathways, offering insights into how certain bacterial genera may contribute to the metabolic issues commonly seen in HT [29]. Our findings indicated higher abundances of several selected bacterial genera in hypothyroid patients; however, because our approach was limited to targeted qPCR of eight genera, no conclusions can be drawn regarding overall gut microbiota richness or diversity.

Ishaq et al. documented significant alterations in gut microbiota composition in HT patients, with notable changes at both the phylum and family levels. Their study identified a reduction in *Prevotella*_9 and *Dialister*​and an increase in the abundance of​ *Escherichia*‐*Shigella* and​ *Parasutterella*. They also reported elevated levels of *E. coli*, indicative of a distinct dysbiosis in HT patients [14]. Similarly, our study observed specific microbial patterns, showing increased levels of certain bacterial types (A bacteria) and decreased levels of others (B bacteria) in hypothyroid patients compared to controls. These findings support the theory that particular bacterial profiles may influence the development or progression of HT.

The concept of the gut‐thyroid axis underscores a complex, reciprocal interaction between gut microbiota and thyroid function, emphasizing the role of gut microbiota composition in shaping thyroid morphology, hormone production, and susceptibility to autoimmune conditions. This axis also includes the regulation of crucial micronutrients like iodine and selenium, essential for maintaining thyroid balance. Through these mechanisms, the gut‐thyroid axis acts as a regulatory pathway with significant implications for endocrine health, immune modulation, and metabolic stability. This intricate relationship suggests that alterations in gut microbiota could have profound effects on thyroid physiology and the risk of thyroid‐related disorders, highlighting the need for further investigation into potential therapeutic approaches targeting the gut microbiome to support thyroid health [30].

Studies have consistently shown differences in gut microbiota composition across various BMI categories, although some results can be contradictory due to diverse study designs and populations [31, 32]. For example, obese individuals often exhibit a higher level of Firmicutes and a lower level of Bacteroidetes compared to lean adults, leading to an increased ratio of Firmicutes to Bacteroidetes (F/B ratio) [32]. This pattern has been also observed in Ukrainian adult populations [33]. Conversely, a study in southern Italy found that overweight participants had lower Firmicutes and higher Bacteroidetes compared to normal‐weight participants, with the F/B ratio inversely associated with BMI [34]. Adult BMI is strongly related to fecal metabolite levels, and numerous associations exist between fecal microbial features and metabolite levels, highlighting the dynamic role of the gut microbiota in metabolism. For example, strong positive correlations between Firmicutes (*Blautia*) and lipid metabolites related to secondary bile acid metabolism were observed in normal‐weight individuals, while obese individuals showed strong positive correlations between Firmicutes (unknown genus) and dicarboxylic acids involved in fatty acid metabolism [35]. In our study, however, BMI did not differ significantly between the hypothyroid and control groups, and median values were broadly comparable. Given the small sample size and non‐normal distribution of the data in this pilot study, formal statistical adjustment for BMI in multivariable models was not methodologically appropriate. Nevertheless, we cannot completely exclude the possibility that subtle differences in body composition, beyond what is captured by BMI alone, may have contributed to the observed microbial differences. The observed variations across studies emphasize the need for large‐scale human studies with diverse populations to systematically describe these associations. Further research, especially longitudinal and interventional studies, is needed to establish causal relationships and explore the potential for targeting the gut microbiota in obesity prevention and treatment strategies [36].

This study has several limitations related to financial and logistical constraints, some of which were exacerbated by the COVID‐19 pandemic, leading to a reduced sample size and imperfect matching by age and sex. Although strict exclusion criteria were applied to control for key confounders such as smoking, recent antibiotic or probiotic use, and chronic comorbidities, the small cohort increases susceptibility to inter‐individual variability. The use of qPCR targeting a limited number of bacterial genera does not capture overall microbial diversity or community structure, which would require 16S rRNA gene sequencing or metagenomic approaches. Moreover, reliance on a single *E. coli* DNA standard does not account for inter‐taxa differences in 16S rRNA gene copy number, and abundance estimates should therefore be interpreted as relative rather than absolute. Low copy numbers observed for *Akkermansia* despite validated primers, uniform extraction protocols, and consistent laboratory conditions may reflect biological scarcity or variability related to sample storage duration and should be interpreted cautiously. Normalization to stool mass, while reducing some variability, does not fully exclude the influence of extraction efficiency or batch effects, particularly in the absence of internal spike‐in controls or copy‐number correction. Additionally, the study groups were drawn from two distinct populations, and incomplete information on HT etiology, disease duration, and levothyroxine dosage limited clinical characterization. A slight gender imbalance and the lack of correction for multiple comparisons further constrain interpretability, rendering the findings hypothesis‐generating rather than confirmatory. Future studies incorporating larger, well‐matched cohorts, internal standards, controlled batch processing, and inclusion of both hypo‐ and hyperthyroid patients are needed to validate and extend these observations.

## 5. Conclusion

This pilot study provides preliminary insights into the association between HT and gut microbiota composition, demonstrating notable shifts in bacterial populations among individuals with HT. Specifically, our findings reveal that hypothyroid patients exhibit a significantly higher abundance of bacterial genera such as *Bacteroides*, *Bifidobacterium*, *Escherichia*, *Fecalibacterium*, and *Prevotella* in comparison to healthy controls. These microbial patterns support the hypothesis that HT may be linked to specific microbial signatures, suggesting a role for the gut‐thyroid axis in the pathogenesis of this condition. To substantiate these findings and further elucidate the precise mechanisms by which gut microbiota might influence thyroid function, future studies should prioritize larger, longitudinal research designs.

## Author Contributions

Zahra Hoseini Tavassol and Farima Farsi contributed equally to this work and are co‐first authors.

## Funding

This study was supported by the National Institute for Medical Research Development (NIMAD) (grant number 983137).

## Ethics Statement

The studies involving human participants were reviewed and approved by the Ethical Committee of the National Institute for Medical Research Development (NIMAD) (ID number: IR.NIMAD.REC.1398.152). The patients/participants provided their written informed consent to participate in this study and for the publication of any potentially identifiable data included in this article.

## Conflicts of Interest

The authors declare no conflicts of interest.

## Data Availability

The data supporting the findings of this study are available from the corresponding author upon reasonable request.
